# From Research-to-Practice: An Adaptation and Dissemination of the COMPASS Program for Home Care Workers

**DOI:** 10.3390/ijerph15122777

**Published:** 2018-12-07

**Authors:** Ryan Olson, Jennifer A. Hess, Kelsey N. Parker, Sharon V. Thompson, Anjali Rameshbabu, Kristy Luther Rhoten, Miguel Marino

**Affiliations:** 1 Oregon Institute of Occupational Health Sciences, Oregon Health & Science University (OHSU), Portland, OR 97239, USA; kelsnparker@gmail.com (K.N.P.); svthomp2@illinois.edu (S.V.T.); rameshba@ohsu.edu (A.R.); luther.kristy@gmail.com (K.L.R.); 2 Oregon Health & Science University (OHSU)-Portland State University School of Public Health, Portland, OR 97239, USA; 3 Department of Psychology, Portland State University, Portland, OR 97201, USA; 4 Labor Education and Research Center, University of Oregon, Eugene, OR 97403, USA; jhessdc@gmail.com; 5 Division of Nutritional Sciences, University of Illinois at Urbana-Champaign, Urbana, IL 61801, USA; 6 Department of Family Medicine, Oregon Health & Science University, Portland, OR 97239, USA; marinom@ohsu.edu

**Keywords:** home care workers, workplace, occupational, safety, health, well-being, dissemination

## Abstract

The COMmunity of Practice And Safety Support (COMPASS) program was developed to prevent injuries and advance the health and well-being of home care workers. The program integrates elements of peer-led social support groups with scripted team-based programs to help workers learn together, solve problems, set goals, make changes, and enrich their supportive professional network. After a successful pilot study and randomized controlled trial, COMPASS was adapted for the Oregon Home Care Commission’s training system for statewide dissemination. The adapted program included fewer total meetings (7 versus 13), an accelerated meeting schedule (every two weeks versus monthly), and a range of other adjustments. The revised approach was piloted with five groups of workers (total *n* = 42) and evaluated with pre- and post-program outcome measures. After further adjustments and planning, the statewide rollout is now in progress. In the adaptation pilot several psychosocial, safety, and health outcomes changed by a similar magnitude relative to the prior randomized controlled trial. Preliminary training evaluation data (*n* = 265) show high mean ratings indicating that workers like the program, find the content useful, and intend to make changes after meetings. Facilitating factors and lessons learned from the project may inform future similar efforts to translate research into practice.

## 1. Introduction

For many Americans, especially older adults, home care workers (HCWs) are a vital source of daily personal support that facilitates their ability to “age in place.” With a growing proportion of aging adults in the US population in coming years, the currently estimated 2.9 million home care and personal care aides is projected to increase by 41% between 2016 and 2026. This growth rate is considerably higher than the 7% average growth for all US occupations [1].

HCWs face job demands that are unique and multi-faceted, and they often lack resources or supports to help them meet such demands [2]. Despite the important service they provide in communities, HCWs remain poorly compensated with a median income of $23,130 that is considerably lower than the median for all US workers [1]. Nearly half the nation’s HCWs rely on low-income tax credits and federal assistance programs to make ends meet [3]. HCWs face a number of physical demands and exposures as they assist older adults with activities of daily living, such as walking and other movements, personal hygiene, dressing, bathing, cooking, and housekeeping. Thus, HCWs often suffer from musculoskeletal strain, are exposed to infectious agents and hazardous chemicals, and are at-risk for puncture injuries from sharps when clients do not discard them properly [4]. Further, because they work alone within the homes of their clients, HCWs lack many occupational safety and health protections that are commonly available for employees in more traditional workplaces (e.g., supervision, environmental safety audits, employer assessment and correction of hazards, co-worker support, safety committees, and safety training) [2]. The degree of worker vulnerability differs for independent contractors compared to those who work for home care agencies, but most all HCWs experience deficits in protections to some degree.

Although HCWs report high satisfaction from the close relationships they develop with clients [4], some home care clients may engage in very challenging behaviors, including verbal and physical aggression. HCWs report incidents of verbal and sexual harassment, which are associated with stress, depression, sleep problems, and burnout [5]. The unique profile of challenges for HCWs points to the critical need for interventions geared toward protecting their safety, health, and well-being.

Research with this socially important workgroup has led to the development of effective interventions to reduce blood and body fluid exposures [6], reduce musculoskeletal pain [7], and improve physical fitness and work ability [8]. Socially supportive group interventions have produced long-term improvements in well-being for family caregivers [9] and improved a range of safety, health, and well-being factors among independent HCWs serving consumer-employers in publicly funded programs in Oregon [10]. In addition to experimentally evaluated programs, there are valuable resources for HCWs developed through participatory methods. For example, the Safe Home Care Project provides resources on safe cleaning and disinfection and on safe practices to reduce risk of injuries from sharps and blood-borne exposures (University of Massachusetts, Lowell, Safe Home Care, n.d. [11]). The Caring for Yourself While Caring for Others handbook (National Institute for Occupational Safety and Health [NIOSH] n.d. [12]) provides a checklist of potentially hazardous work tasks, along with tips and tools for preventing exposures and injuries for each family of tasks. The handbook also addresses communication strategies and workplace stress. Helpful illustrations show workers examples and non-examples of safe practices and tool use, and overall, the book is designed to help facilitate conversations between workers and their clients (or with their family members or workplace supervisors) about improving workplace safety.

### 1.1. Translating Evidence-Based Interventions into Practice

In the healthcare domain, Balas and Boren [13] stated that “…it takes an average of 17 years for research evidence to reach clinical practice” (p. 66). For nine clinical procedures established effective in landmark trials, the authors reported current rates of procedure use between 17.0% to 70.4%. Even in medicine, there are very long time lines to realize variable degrees of adoption. 

Typically, an intervention’s reach beyond the research setting is limited by constraints such as a lack of funding to facilitate its usability and scalability or the absence of structures or partners to market it to potential adopters. In some cases, an intervention’s features may not encourage its transfer to practice (e.g., too complex, costly, or effortful to implement). Moreover, intervention researchers are typically evaluated and rewarded for obtaining competitive grants, conducting innovative and high quality research, and publishing research findings. Occupational incentives are not typically aligned for investigators to adapt, commercialize, market, and disseminate the evidence-based programs they create or study. These types of barriers include the overarching culture of peer review, which tends to emphasize factors related to the internal validity of intervention studies over issues related to external validity (e.g., factors that influence participation, adoption, and implementation). To illustrate, in a review of health promotion intervention research using the widely recognized RE-AIM framework, Bull and colleagues [14] reported that just 25% of studies reported information on adoption, and only 12.5% reported implementation data.

In order to overcome some of these barriers, Harris and colleagues [15] proposed a dissemination framework that addresses the gap between scientists and end users of evidence-based health promotion interventions. Their approach relies on a motivated “disseminator” or intermediary organization to help researchers adapt, market, and disseminate interventions. In their model, researchers and intermediary disseminating organizations form a reciprocal relationship that generates “Dissemination Resources” that are then marketed to end users—primarily by the disseminating organization. The authors provided two examples of interventions that were successfully disseminated through this approach that had reached nearly 2000 employers and community based adopters at the time of the publication.

Systematic reviews of research on the translation of community-based interventions into practice suggest additional facilitators for success. Matthews and colleagues [16] shared that translating physical activity interventions into practice was facilitated by tailoring an intervention to suit the intended adopter, partnering strategically with receptive organizations, and adequately training implementers. In another review, Estabrooks and Glasgow [17] shared that interventions are more translation-friendly if they are perceived by the intended users as being more advantageous than existing practices, compatible with their current needs and values, feasible to deliver and implement, able to be tested for potential adoption, and have demonstrated effectiveness among stakeholders [18].

Other dissemination research highlights the importance of commitment from organizational leaders (including financial support) and the presence of workplace champions. The successful large-scale adoption of the evidence-based Stand Up Australia intervention (disseminated as the BeUpstanding Program^TM^) was attributed by researchers in part to a strong partnership with and timely funding support from the adopter (government, in this case). The authors also reported the importance of packaging the intervention into an online toolkit, and then transferring the toolkit to a workplace champion. The toolkit helped champions by providing practical strategies for making a business case for the intervention, obtaining buy-in from organizational leaders, and how to deliver and evaluate the program [19]. Qualitative research with adopters and non-adopters of the evidence-based PHLAME wellness program for firefighters highlighted the importance of a committed chief and the presence of a workplace wellness champion at adopting fire stations (i.e., the “champ-and-chief” model of adoption) [20].

### 1.2. Translating Home Care Worker Interventions for Practice

Although evidence-based and useful interventions for HCWs and other caregivers are available, we were unable to find any peer-reviewed publications regarding their dissemination. Therefore, we reached out for phone interviews with contacts for caregiver programs or resources we were familiar with. The principal investigator for the Safe Home Care Project (M. Quinn, personal communication, 5 October 2018) indicated that its dissemination occurs via fact sheets containing safety and health education, publications in scientific journals, and articles within HCW trade association magazines. The Health Education Program, a supportive group program for family caregivers, is distributed by the principal investigator when requested by caregiver agencies (R. Toseland, 26 September 2018). The Caring For Yourself While Caring For Others handbook, developed by the Labor Occupational Health Program in partnership with NIOSH for Alameda County workers in California, was later adopted within the Alameda County Health Consortium. However, its current dissemination channels outside of being downloadable through its website are unknown (L. Stock, 26 September 2018). In these selected cases, translation, dissemination, and adoption efforts have not been systematically described, and appear to have been supported by dedicated principal investigators or partners without funding or formal systemic support following initial development and evaluation research.

In our view, an important way to address this gap and inform future translation and dissemination efforts, is to publish qualitative and quantitative descriptions of barriers, facilitators, and successes in dissemination after interventions are established to be effective. On this theme, the current paper reports the process and results of a successful intervention adaptation and dissemination effort in progress. The COMmunity of Practice And Safety Support (COMPAS*S*) program was designed to advance the safety, health, and well-being of HCWs. The intervention’s peer-led and supportive group tactics are aimed at creating a protective occupational social support structure for typically isolated HCWs. Developed using a *Total Worker Health*^®^ approach, COMPASS simultaneously focused on reducing hazards for injuries and illnesses, while also promoting factors to advance workers’ health and well-being. The intervention was developed with labor and governmental partners, and was demonstrated effective through a cluster randomized controlled trial (RCT). A range of factors showed significant improvements, including workers’ experienced community of practice, safety behaviors (including ergonomic tool use), and health factors (such as fruit and vegetable consumption [10]). Following the RCT, COMPASS was adapted for dissemination with the Oregon Home Care Commission (OHCC), pilot tested in its adapted form, and is now being rolled out statewide by the OHCC as a paid training opportunity available to roughly 60% of Oregon’s home care workforce. The current project describes original and adapted programs, results from the pilot test of the adapted program, and preliminary training evaluation data from the COMPASS rollout by the OHCC.

## 2. Materials and Methods

The COMPASS research program began formally in 2011 as a research project within the Oregon Healthy Workforce Center, a Center of Excellence in *Total Worker Health*^®^ (NIOSH U19OH010154). Over the life of the program, all COMPASS research procedures have been reviewed and approved by the Oregon Health & Science University Human Subjects Institutional Review Board. Research partners include the Service Employees International Union Local 503 and the OHCC. After supporting initial development and evaluation research, both the union and the OHCC continued support for the intervention’s translation into practice, with the OHCC as the ultimate adopting organization. The OHCC is housed within Oregon’s Department of Human Services as a component of state-supported Services for Seniors and People with Disabilities. The OHCC is charged with defining qualifications for HCWs and other caregivers who provide services for consumers who qualify for publicly funded care. These HCWs work as independent contractors without a supporting home care agency, supervisor, or co-workers, and are employed directly by their clients who are referred to as “consumer-employers.” The OHCC operates a training system offering over 20 course topics for HCWs and personal support workers throughout the state, manages a registry to match workers with consumer-employers (or consumer-employers with workers), and serves as the employer of record for collective bargaining with the union.

The original three-year long COMPASS research project [21] included intervention development followed by a pilot study [22] and cluster RCT [10]. The original trial design involved baseline, 6 month, and 12 month measurements. Participants were recruited from among the population of HCWs caring for consumer-employers who qualified for publicly funded home care services through the OHCC managed system. As the RCT was underway, the Oregon Healthy Workforce Center applied for and was awarded an additional two years of funding. In that extended two-year agenda an additional follow-up measurement was added (≈24 months post-baseline), as well as qualitative research focused on caregivers’ experiences at work and in the COMPASS program [2]. Further research was planned to conduct interviews with leaders and workers at private home care agencies to inform future dissemination in that industry segment. However, when the opportunity arose to adapt and potentially disseminate COMPASS within the OHCC training system, dissemination aims with private agencies were postponed and the intervention was adapted and piloted for dissemination in the Commission’s training program. Translation and dissemination efforts continued after research grant funding for COMPASS ended in 2016. Further adaptation of the intervention materials and process was completed with financial support from the Commission and the Oregon Institute of Occupational Health Sciences at Oregon Health & Science University. In the Fall of 2017 COMPASS was added to the OHCC’s training system as a paid course offering for workers. Below we describe the methods for each phase of the COMPASS research with an emphasis on how intervention materials and processes were adapted, piloted, and translated into practice.

### 2.1. COMPASS Pilot and Randomized Controlled Trial

The original COMPASS intervention development and evaluation has been described in previous publications in detail. However, a high-level summary is needed to understand how materials and processes were modified for translation into practice. COMPASS is a supportive group program that is peer-led and scripted. Intervention tactics employed were modeled on effective social support groups [9,23,24] and scripted team-based health promotion programs [25,26,27,28]. Original intervention resources developed included two types of guidebooks: a group leader guidebook with additional instructions and activity answers, and a group member guidebook without those group-leader specific instructions and activity answers. In both the pilot and RCT, groups met monthly to complete a scripted meeting that followed a ritualized structure. Each meeting involved a WorkLife check-in, educational lesson, goal setting and follow-up, a healthy meal break, and a WorkLife support activity.

The curriculum was developed in two phases so the RCT could begin while investigators continued to develop and pilot test additional scripted meetings. In the first phase, leader and member versions of guidebook one were developed with seven scripted meetings that were evaluated by workers (*n* = 16) in the published pilot study, and then revised and evaluated in the first half of the RCT intervention phase (16 clusters of workers, *n* = 149). In addition to the scripted meetings, guidebook one included an “Extras” section with additional resources for workers, including the Gershon Home Hazard Checklist, the NIOSH Caring for Yourself While Caring for Others handbook, and perforated cards printed with templates for behavioral self-monitoring activities. Pages for tracking attendance and goals were also included. In an unpublished phase of the pilot study, guidebook two (six additional meetings) was developed and then evaluated with a subgroup of pilot participants (*n* ≈ 6) as the RCT began using guidebook one. Guidebook two was also modified based on pilot results, and then used in the second half of the RCT intervention phase. The second guidebook included a slightly different structure. In the educational lesson component, groups chose from a menu of homework reading topic options to read before the next meeting; these topics were then discussed at the next meeting using a structured discussion guide. Like the first guidebook, the second guidebook included forms for tracking attendance and goal completion and perforated cards printed with templates for behavioral self-monitoring activities.

Seven of the eight original RCT intervention groups were led by a permanent peer-leader who was involved in the published pilot study. The eighth group lacked a peer-leader from the pilot, and was therefore led by two peer co-leaders who volunteered for this role at their group’s first meeting. Guidebook materials were supported with a peer leader toolkit of ergonomic tools and objects for activities (slide boards, transfer belts, anti-friction disks, Gimme-A-Lift, and tennis balls), as well as resource giveaways used for completing “take home goals” (knee pads, step counters, and wrist bead counters for behavioral self-monitoring). Workers also received pay and incentives for attending meetings and research data collection waves, and were recognized with participation-based incentives for earning individual and/or group certification. During the first six months, participants obtained individual certification if they attended five team meetings and completed five individual goals; teams were certified if the entire team completed five team goals. During the second six months individual certification was earned if the participant attended at least four meetings and completed eight individual goals (one repeat goal and one new goal were selected each meeting); team certification was awarded if all team members completed four or more team goals. Those who earned individual certification obtained a $60 gift certificate and certified teams received COMPASS jackets (first six months) and COMPASS umbrellas or a team patch (second six months). Participants were paid $11 an hour through the grant for study-related activities prior to October 2013, and $15 an hour thereafter following a state-wide wage increase. Supplemental incentives included a $30 retention bonus at follow-up research data collection periods, lottery drawings for supplemental compensation awards totaling $1000 (many small awards were drawn), and additional gifts at baseline (COMPASS tote bag), 6 months (COMPASS t-shirt), and 12 months (COMPASS lunch bag).

### 2.2. COMPASS Adaptation and Pilot for the OHCC

During the development and research phases for COMPASS investigators solicited union and OHCC input, provided regular updates to the OHCC training committee and the union to inform them of progress and findings, and periodically discussed the future of the program. Following the successful RCT, and in response to ongoing dialogue about the program, the OHCC requested that investigators adapt COMPASS to be offered as a paid training course in their system. One motivation for this decision expressed by OHCC leaders was an interest in cultivating leadership skills among HCWs. A plan for adapting COMPASS guidebook one was worked out collaboratively, implemented by researchers, and then pilot tested. Adjustments to the approach were guided by OHCC practical needs, but with a commitment to retaining core evidence-based tactics. Adjustments included: a faster cycle time, where groups met every other week instead of once a month; reduction of total meetings to seven (e.g., only topics from guidebook one); the use of professional OHCC-contracted trainers to serve as group facilitators who would lead the first meeting and then support each group as a “guide on the side” thereafter; using rotating volunteer peer-leaders at meetings two through seven; removing the meal served during breaks; replacing some original group and individual goals to attend related OHCC trainings with new goals focused on workers making targeted work-environment and behavior changes; and incentive adjustments. The long-term strategic plan also included proposed adjustments to training evaluation questions for all OHCC training courses in order to accommodate a peer-led course series like COMPASS. Table 1 summarizes COMPASS guidebook one original topics and goals, as well as adaptations (most adaptations were made prior to the adaptation pilot, but some were made afterward).

The adaptation pilot involved five COMPASS groups led by four OHCC facilitators in three Oregon cities. This sampling approach was selected to provide a check that the adjusted process was functional across multiple facilitators and groups, and that the intervention was changing targeted outcomes by a similar magnitude (effect size) relative to the effective version evaluated in the RCT. Workers received hourly wages for attending COMPASS adaptation pilot meetings, just as they would for attending other courses offered by the OHCC (workers receive wages for any non-duplicated course annually). Pilot participants received an additional $15 for completing surveys and/or taking part in an interview with study staff. Plans were also made for COMPASS to satisfy safety training requirements (completion of two safety courses every two years) for workers to be listed on the OHCC registry for finding (or being found by) potential new Medicaid/Medicare-funded consumer-employers. The adapted program was supported with the same ergonomic toolkit and resource giveaways as the intervention as implemented in the RCT. However, no incentives were provided for individual or group certification. Instead, printed paper certificates were awarded based on attendance. As noted above, professional trainers under contract with the OHCC were identified to serve as COMPASS facilitators. The research team created a half-day orientation and facilitator training workshop to prepare facilitators for their role. This training included a history of the program and research findings, description and handouts on the role of facilitators, and practice with scripted guidebook activities with coaching from researchers. Guidebooks and other materials for implementation were provided to facilitators before their first group meeting.

At baseline researchers collected direct measures of height (SECA 213 stadiometer; SECA, Chino, CA, USA) and weight (Tanita TBF-310GS; Tanita Corp, Arlington Heights, IL, USA), and survey measures of demographics, work history, and current work characteristics. Pre- and post-program evaluation measures emphasized outcomes from the prior RCT [10]. Survey scales/items included experienced community of practice [29]; frequency counts for five types of safety behaviors [10]; fruit and vegetable consumption (single item 1–10+ servings daily, created for adaptation pilot); consumption of sugary drinks, snacks, and fast food meals [30]; frequency of meals brought from home [30]; weekly healthy physical activity levels [26]; and physical and psychological well-being [31].

### 2.3. Statewide Rollout of COMPASS in the OHCC Training System

In parallel with and following the adaptation pilot test, several efforts were initiated to support eventual adoption and statewide rollout of COMPASS within the OHCC’s training system. These efforts included initiating negotiations for an interagency agreement between Oregon Health & Science University (OHSU) and the Oregon Department of Human Services to govern the terms of use of guidebooks; investigators requesting adjustments to the standard OHCC training evaluation questions to accommodate a course series like COMPASS; and revisions to the OHCC version of the COMPASS guidebook in response to observing the pilot and in response to guidance from OHSU Technology Transfer and Business Development. We also explored whether other stakeholders, such as the relevant workers’ compensation insurer or an SEIU Health Trust, would support or fund parts of the dissemination effort. These conversations did not result in direct financial support for dissemination efforts, but helped guide sustainability decisions and resulted in in the addition of information about an Employee Assistance Program available to HCWs to the Extras section of COMPASS guidebooks.

Negotiations for terms of use of the program took quite a long time, in part due to the timing of the retirement of the OHCC’s Training Director and other staff turnover. Other hurdles involved navigating unclear review and approval steps within the state government, and some long inter-agency response times for document review requests. At the conclusion of over a year of episodic back-and-forth work and hand-offs on the inter-agency research agreement, the ultimate terms granted the OHCC non-exclusive rights to print and use COMPASS guidebooks in exchange for sharing long-term evaluation data with OHSU (5+ years). Evaluation data would include class attendance for COMPASS and non-COMPASS courses, class evaluations, and assisting OHSU in coordinating with the workers’ compensation insurer to obtain injury claims data for workers who took COMPASS over the years and cross-sectional comparison groups of workers who either had not taken any training, and those who had taken some training classes (but not COMPASS).

Within the OHCC training system, at the end of each class HCWs are asked to complete a training evaluation (no names recorded) and leave it for their trainer to collect. Some of these original evaluation items did not clearly apply to a peer-led and scripted program. For example, one item asked workers to rate the degree to which “Information was presented in a variety of ways to facilitate learning.” In COMPASS, all of the material is presented using a single scripted and peer-led method, and past research showed that this method produced large knowledge gains [22]. Two additional items asked students to rate the trainer’s performance (preparedness and communication effectiveness), but in COMPASS, there is a supporting facilitator rather than a traditional trainer. In addition to recommending adjustments to questions like those above, researchers also requested additional questions that asked workers to rate their intentions to make changes as a result of the training.

To further strengthen the program and streamline dissemination, COMPASS guidebooks were further adjusted in ways to support their use in the OHCC training system. Giveaways for students that had no funding stream within the state (or potential funding stream) to support their use in the OHCC version of COMPASS, and homework assignments facilitated with such giveaways (e.g., small knee pads), were removed from the program. Investigators also replaced some activities with new ones, and made adjustments to other activities to make them run more smoothly. With guidance from OHSU Technology Transfer and Business Development, investigators also worked with an OHSU graphic design specialist to create a professional design and layout for the guidebooks.

After the agreement was settled and signed, and the revised OHCC guidebooks with the new design were ready, the OHCC and investigators planned a “soft launch” of COMPASS by two facilitators in two cities. One facilitator had participated in the original adaptation pilot, and the other was new to the COMPASS program. The new facilitator and other OHCC staff received in-person training from investigators regarding COMPASS group facilitation, and the facilitator with previous experience had a meeting with investigators to be refreshed on the program and be informed of changes and adjustments made. Following the soft launch further technical corrections were made to the guidebooks (fixing typos, clarifying arrangements) and we removed all remaining giveaways for students and replaced them with alternatives (e.g., step counter giveaways used for walking challenges were removed, and activities/goals were altered to focus on “walking minutes per day/week”). As of October 2018, 2 additional facilitators were trained by OHCC training department staff and 12 groups have been completed or initiated in 7 different cities.

## 3. Results

### 3.1. Adaptation Pilot Results

Four facilitators (*n* = 3 female) were recommended by the OHCC Training Director and trained by investigators for the adaption pilot. Forty-two home care workers registered for five COMPASS groups (one facilitator ran two groups) offered in the following cities: Albany (*k* = 2), Salem (*k* = 2), and Corvallis (*k* = 1). The groups were offered at varied times to maximize opportunities for workers, with one in the late morning, three in the early afternoon, and one in the evening.

Participants were predominantly older (mean age = 49.23 years), female (80.56%), and Caucasian (77.78%). Workers reported an average tenure in home care of 7.12 years and an average of 22.37 weekly work hours. The reported lifetime prevalence of a diagnosis of depression or anxiety was 41.67% and 44.44%, respectively. For additional demographic details please see Table 2.

Changes in primary outcomes were evaluated with descriptive effect sizes (Cohen’s *d*) and two-tailed *t* tests. Given that the pilot was designed to evaluate feasibility and check effect sizes, and not to be a fully statistically powered effectiveness study, inferential *t*-test results should be viewed as supplementary and interpreted with the understanding that type II errors were probable. Pre- and post-program means, mean changes, effect sizes, and *p* values are reported in Table 3. Moderate-to-large effect sizes and statistically significant changes were observed for experienced community of practice, using new tools/techniques for housecleaning, fruit and vegetable consumption, meals brought from home, and healthy physical activity. All other outcomes changed in expected directions with the exception of two safety outcomes that had very small negative effect sizes (<0.10).

### 3.2. Statewide Rollout: Additional Adaptations in Progress and Preliminary Training Evaluation Results

As the statewide rollout of COMPASS was being planned and initiated, the OHCC expressed interest in further adaptations being made to COMPASS so that it would be inclusive of personal support workers. Within Oregon’s publicly funded programs, personal support workers provide care for individuals with cognitive and developmental disabilities who qualify for publicly funded support services. Service recipients’ ages range across the lifespan. This type of work was viewed to share many similarities with home care work, but also pose some different safety, health, well-being demands that might suggest new or revised guidebook activities and content. Investigators contracted with the OHCC to conduct formative research with personal support workers (e.g., survey, qualitative interviews) and make tailored curriculum adjustments based on findings. Bundled in this new phase of dissemination work included the development of interactive online orientation training for new COMPASS facilitators and creating promotional videos for the program. It is anticipated that after COMPASS is revised to accommodate personal support workers, the OHCC will then translate the guidebooks into several non-English languages to encourage non-native English speakers to participate in higher numbers.

As noted in the methods section, 12 COMPASS groups have been completed or are in progress in the statewide rollout. For the current analysis, the OHCC provided training evaluations (*n* = 265) from workers who participated in COMPASS group meetings from summer 2017 to summer 2018. Two different training evaluation question sets were completed by workers in the data set. An older version (but more recent than the original OHCC training evaluation questions prior to the adaptation pilot) was completed by 229 workers. A newer version, adjusted to better accommodate evaluations of the COMPASS dissemination, was completed by 36 workers. Table 4 provides mean ratings and standard deviations for the older evaluation questions for each COMPASS meeting, as well as an overall average rating for each question across meetings. All mean ratings were above 4 (max = 5) on a scale where 1 = poor, 2 = fair, 3 = average, 4 = good, and 5 = excellent. Table 5 reviews mean ratings and standard deviations for each question in the new format, but not for each meeting due to insufficient data. For those questions mean ratings all averaged above 3 (max = 4) on a scale where 1 = strongly disagree, 2 = disagree, 3 = agree, and 4 = strongly agree. Attendance data and evaluation data for other comparable OHCC classes are not yet available for analysis.

## 4. Discussion

The current project represents a rare, and thus far successful, effort to translate an evidence-based intervention into practice for HCWs. A pilot test of the adapted program confirmed that adjusted intervention processes were functional, and that outcomes were changing with effect sizes resembling those from the prior randomized controlled trial. Preliminary evaluation data suggest the program is well liked, rated as useful, and that workers report intentions to make safety and health changes as a result of participating. An additional strength of the dissemination effort includes an inter-agency research agreement that will generate long-term evaluation data for COMPASS participants (attendance, course evaluations, workers’ compensation injury claims) with potential for some cross-sectional analyses of HCWs within the state. Several factors facilitated this successful dissemination effort and its probable long-term sustainability, but key factors included engagement and collaboration with the adopting partner (the OHCC) in initial intervention development, continued dialogue and discussion during research phases, flexibility from both investigators and the OHCC while adjusting the program to meet training system needs, and sustained top level commitment at the OHCC. Other researchers may consider following a similar model of sustained partnership across research phases in order to enhance a sense of ownership from the adopting partner and promote dissemination sustainability. Considering the factors Rogers [18] pointed out as being important for successful dissemination, we believe the OHCC perceived COMPASS as compatible with their current needs and values, feasible to deliver and implement, able to be tested for potential adoption (e.g., our adaptation pilot), and to have demonstrated effectiveness among stakeholders (this included early and consistent collaboration with and commitment from the union). Consistent with dissemination research findings of Kuehl et al. [20], leadership support included both committed chiefs (the Executive Director of the OHCC, Senior Director of Technology Transfer at OHSU) and champions (OHCC Training Directors and key members of the OHCC Training Committee).

While many facilitating factors were present, the dissemination effort faced some barriers to success. Some of these barriers included turnover of key staff at various time points, gaps in funding, and complexities in navigating government and university processes. Into the future, the long-term success of the adoption of COMPASS in the OHCC will depend on some level of continued support from OHSU, the Oregon Institute of Occupational Health Sciences, and the Oregon Healthy Workforce Center (e.g., to update and adapt the curriculum) as well as continued financial commitment from the OHCC/state of Oregon to fund guidebook printing and wages for facilitators and workers. Stable state funding for both the OHCC and the Oregon Institute of Occupational Health Sciences will be key, as well as continued NIOSH funding for the Oregon Healthy Workforce Center. Thus, investigators and partners at the OHCC will need to plan ahead, anticipating courses of action if any one of these sources of support is disrupted. This includes evolving structures at the Oregon Institute of Occupational Health Sciences to support translation and dissemination of evidence-based programs created by its investigators and their collaborators.

Looking outside of the state of Oregon in the US, we recommend that researchers who aim to develop, evaluate, and disseminate interventions for similar populations investigate institutional structures and potential partners within their target region. For publicly funded HCWs in Oregon, both union and governmental partners were equally engaged through intervention development and evaluation phases, but governmental partners played the most central role in the dissemination phase because of their ownership of the training system for publicly funded HCWs. In contrast, in our neighboring state of Washington, an SEIU 775 Benefits Group “owns” and operates training programs for publicly funded HCWs. Therefore, development and evaluation phases of an intervention for HCWs in Washington would similarly need to involve both government and labor partners, but during a dissemination phase, the union benefits group would play the most central collaborative role.

Given that the literature on translation of *Total Worker Health*^®^ interventions into practice is in its infancy, future dissemination and implementation science with effective programs is strongly encouraged. For example, if interest in COMPASS expanded and resources are available to study a new dissemination effort, research could systematically evaluate organizational readiness factors and measure adoption and implementation of the program across multiple new organizations. Such future dissemination science with any particular *Total Worker Health*^®^-informed intervention may be fruitfully guided by recommendations and tools from Dugan and Punnett [32]. Based on their experience developing the Healthy Workplace Participatory Program, the authors provide examples of potential dissemination and implementation studies and tools, such as the Five and Ten D&I Evaluation Tool to assess specific implementation outcomes.

## 5. Conclusions

HCWs are a priority population for protective interventions that advance their safety, health, and well-being. Rapidly growing in number, HCWs help some of our most vulnerable citizens remain in their homes and enjoy a higher quality of life. In-home care may also create critical cost-savings in the healthcare system by preventing older adults from transitioning prematurely to long-term care facilities. While some interventions have research evidence for their effectiveness, and other valuable tools and resources are available, we could not find a descriptive or empirical paper about a successful research-to-practice intervention dissemination effort with HCWs. In this regard, the current description of the adaptation and dissemination of COMPASS with the OHCC may help guide future similar efforts with other partners or in other states. Consistent with previous findings in the dissemination science literature, the success of the current effort was facilitated by several favorable factors that can be cultivated in future projects by other intervention scientists. These include early engagement and collaboration with industry and labor in the development of interventions, designing for feasibility and repeatability of intervention tactics, and sustained engagement over time to foster strong relationships and top-level leadership commitment over the long term. We also believe flexibility and persistence from all parties was essential for overcoming barriers as they arose. This persistence included a commitment to the effort during times when resources for dissemination efforts were scarce or uncertain. We are optimistic with continued contingency and structural planning that the rollout of COMPASS will be sustained within the OHCC, with the ultimate potential to improve the safety, health, and well-being of approximately 60% of the Oregon home care work force.

## Figures and Tables

**Table 1:** COMmunity of Practice And Safety Support (COMPASS) guidebook one: Topics with original and adapted goal options (adaptations are in ***bold italics***).

Meeting and Topic	Content and/or Sample Activities	Original Goals	Adjusted Goals (as of Fall 2018)
1. Team Building Workshop	Team building activities How safety and health are related How COMPASS teams work	Group Goal Odds and Evens Step Challenge Individual Goal Options Watch a “23 and ½ h” video Daily activity tracking with app Take an OHCC class on any topic	Group Goal Odds and Evens ***Walking Minutes*** Challenge Individual Goal Options ***Aim for 30 min daily walking via:*** ***Workday walks*** ***Errand/commute walks*** ***Free time walks***
2. Fruits & Vegetables ***More Plants on the Plate***	Fruit and vegetable serving sizes and recommendations Nutrition information and game(s) ***Harvard Healthy Eating Plate and whole foods***	Group Goal Bring healthy recipe to share with group Individual Goal Options Track fruit and veggie servings and strive for 5 daily Try four new fruits or veggies or prepare them in a new way Swap out sugary drinks and replace with water or zero calorie drinks	Group Goal ***Fruit and veggie challenge to track and eat 5 servings daily for two weeks*** Individual Goal Options ***Fill half of plate at meals with fruits and vegetables*** ***Swap out high-calorie snacks and replace with fruit or veggie snacks*** Swap out sugary drinks and replace with water or zero calorie drinks
3. Back to Healthy Postures	Practice finding neutral spine Tips for maintaining neutral spine during common activities	Group Goal Track neutral spine postures by task or alarm Individual Goal Options Track neutral spine by alarm Track neutral spine by task Attend OHCC class titled “Protecting Against Sprains and Strains”	Group Goal ***Identify a housekeeping task and do in a new way that protects neutral spine posture*** Individual Goal Options Track neutral spine by task Track neutral spine by alarm ***Deleted OHCC course goal***
4. Functional Fitness	Core strength and practical/functional fitness Anywhere core exercises Healthy Hobbies	Group Goal Repeat Odds and Evens Step Challenge Individual Goal Options Core exercise scavenger hunt Pair with group member and do a healthy hobby activity Find and take part in an exercise class/resource in the community	Group Goal Repeat Odds and Evens ***Walking Minutes*** Challenge Individual Goal Options Core exercise scavenger hunt ***Strength training twice a week*** Find and take part in an exercise class/resource in the community
5. Take a Load Off With Tools	Safety traps that lead to injuries Common injuries Low tech tool introduction and practice	Group Goal Complete the “Gershon Home Hazard Checklist” in consumer-employers’ homes Individual Goal Options Attend relevant OHCC class like “Durable Medical Equipment” or “Protect Against Sprains and Strains” Research tools online or at a medical supply store Watch video on Gimme-a-Lift or Slide Boards with Transfer Belts	Group Goal ***Complete the NIOSH “Caring for Yourself While Caring for Others” hazard checklist*** Individual Goal Options ***Increase use of tools already on hand*** ***Use the “Caring for Yourself While Caring for Others” booklet to identify tools needed*** ***Talk to a case manager about needed tools or training***
6. Communicating for Hazard Correction	Learn effective and less effective ways to communicate about hazards Learn PRAISE mnemonic communication strategy Role play communication with consumer-employer	Group Goal Use the NIOSH “Caring for Yourself While Caring for Others” booklet and discuss relevant hazards with your consumer-employer Individual Goal Options Good day/bad day interview with consumer-employer Attend OHCC class on communication such as “Challenging Behaviors”, Keeping it Professional”, or “Working Together” Use PRAISE strategy with others	Group Goal Discuss relevant hazards with your consumer-employer ***that you identified previously using the NIOSH “Caring for Yourself While Caring for Others” booklet*** Individual Goal Options Good day/bad day interview with consumer-employer Use PRAISE ***skills with a friend or co-worker*** ***Find and correct a hazard in your own home***
7. Mental Health	Practice guided relaxation Three good things activity ***Positive/negative talk activity and healthy stress coping***	Group Goal Set a personal safety or health goal from any area COMPASS covered Individual Goal Options Gratitude journal Progressive muscle relaxation daily for one week Attend OHCC class “Stress Management and Relaxation Techniques”	Group Goal ***COMPASS mental health book club*** Individual Goal Options Gratitude journal Progressive muscle relaxation daily for one week ***Track stressful events and practice healthy stress coping strategies learned in COMPASS***

Note: Guidebook adjustments are noted with ***bold italics.*** NIOSH: National Institute for Occupational Safety and Health; OHCC: Oregon Home Care Commission. All goals had “short hand” names for workers to remember and name their choice easily, but in the table, are written out in descriptive form. In the randomized trial version of COMPASS, groups used a poster with the image of a house with doors and windows to track group and individual goal completion. In the adapted version of COMPASS the poster was not used due to removal of the certification incentives (a simple attendance requirement for getting COMPASS safety class credits was instituted instead). The PRAISE mnemonic was created by Dr. Robert Wright, and stood for: Plan, Respect, Ask open ended questions, use “I” statements, and Express empathy.

**Table 2:** Home care worker participants in the pilot study of the COMPASS adaptation for the Oregon Home Care Commission: Characteristics at baseline.

Measure	*n*	OHCC-Pilot Sample *n* = 36 ^a^
Age, Mean (*SD*)	35	49.23 (12.16)
Female, *n* (%)	36	29 (80.56)
BMI Mean (*SD*)	35	31.38 (7.41)
Race, *n* (%)	36	
Caucasian		28 (77.78)
American Indian/Alaskan Native		1 (2.78)
Asian		1 (2.78)
Native Hawaiian/Pacific Islander		1 (2.78)
Black/African American		0 (0.00)
More than one race		3 (8.33)
Other		2 (5.56)
Relationship status, *n* (%) ^b^	35	
Married		19 (54.29)
Divorced/Separated		8 (22.86)
Living with Sig. Other		4 (11.45)
Never Married		4 (11.45)
Highest degree completed, *n* (%)	35	
No certificate or degree		2 (5.71)
High School Diploma		8 (22.86)
Vocational/Tech. Certificate		5 (14.29)
Associates Degree		6 (17.14)
College Degree		11 (31.43)
Graduate School Degree		3 (8.57)
Tenure as home care worker	36	
Mean (*SD*)		7.12 (7.92)
Range		0.25–38.00
Daily work hours	19	
Mean (*SD*)		8.74 (7.24)
Range		2.00–24.00
Weekly work hours	29	
Mean (*SD*)		22.37 (17.25)
Range		2.00–64.00
Number of public consumer-employers ^c^	29	
Mean (*SD*)		1.72 (1.03)
Range		0.00–4.00
Number of private consumer-employers	13	
Mean (*SD*)		0.77 (0.60)
Range		0.00–2.00
Number of dependent children	36	
Mean (*SD*)		0.36 (0.83)
Range		0.00–4.00
Ever diagnosed w/ depression, *n* (%)	36	15 (41.67)
If yes, taking meds, *n* (%)		7 (46.67)
Ever diagnosed w/ anxiety, *n* (%)	36	16 (44.44)
If yes, taking meds, *n* (%) ^d^		7 (43.75)
Ever diagnosed w/ chronic pain, *n* (%)	36	8 (22.22)
If yes, taking meds, *n* (%) ^e^		4 (50.00)
Ever diagnosed w/ diabetes, *n* (%)		9 (25.00)
If yes, taking meds, *n* (%)		7 (77.78)
Ever diagnosed w/ hypertension, *n* (%)	36	11 (30.56)
If yes, taking meds, *n* (%) ^f^		9 (81.82)

Note: OHCC = Oregon Home Care Commission. ^a^ This sample size represents workers who enrolled at baseline and returned for post-intervention measurements. When percentages are reported they reflect the percent of those reporting for that variable. ^b^ The survey failed to provide an option for participants to select “single”, or to set a time frame for the recency of divorce or separation status. ^c^ An outlier data point of 78 reported current public consumer-employers was removed for analysis of this variable due to the improbability that such a number could be correct. ^d^ 3 did not report yes or no for medication. ^e^ 1 did not report yes or no for medication. ^f^ 1 did not report yes or no for medication.

**Table 3:** COMPASS adaptation pilot intervention effects on primary outcomes.

Primary Outcomes (Time Anchor)	*N*	Pre Mean (*SD*)	Post Mean (*SD*)	Mean Effect	Effect Size (*d*)	*p*-Value
Experienced community of practice ^a^ (3 mo)	35	35.56 (5.03)	39.67 (4.48)	+4.11	+0.86	<0.000
Safety Behaviors ^b^ (3 mo)						
Talked with CE about improving unsafe conditions	34	2.15 (1.71)	2.08 (1.73)	−0.07	−0.04	0.865
Corrected slip/trip/fall hazards	34	1.62 (1.44)	1.50 (1.38)	−0.12	−0.09	0.714
Corrected other hazards	34	0.91 (1.00)	0.71 (1.00)	−0.20	−0.20	0.292
Used new tool/techniques for moving objects or CEs	35	1.31 (1.47)	1.43 (0.96)	+0.12	+0.10	0.701
Used new tools/techniques for housecleaning	35	1.11 (0.96)	1.69 (0.99)	+0.58	+0.59	0.009
Daily Diet/Exercise Behaviors (1 mo)						
Fruit & vegetable servings	35	4.05 (1.71)	4.87 (1.60)	+0.82	+0.49	0.026
Sugary snacks ^c^	35	3.91 (1.87)	3.20 (1.55)	−0.71	−0.42	0.865
Sugary drinks ^c^	35	3.14 (2.33)	2.51 (1.60)	−0.63	−0.32	0.714
Fast food ^c^	35	2.14 (1.09)	1.97 (1.01)	−0.17	−0.16	0.292
Meals brought from home ^c^	35	5.18 (2.39)	6.14 (2.70)	+0.96	+0.38	0.009
Healthy physical activity ^d^	34	2.44 (1.52)	3.65 (1.58)	+1.21	+0.78	<0.000
Well-Being (1 mo)						
SF-12 physical composite score	31	48.05 (11.51)	48.38 (8.23)	+0.33	+0.03	0.834
SF-12 mental composite score	31	50.37 (10.17)	51.18 (6.75)	+0.81	+0.10	0.699

Note: Sample size varied due to missing responses for certain questions. Cohen’s *d* effect sizes were computed using the pooled standard deviation for pre and post-test time points. *p*-values are for two tailed *t*-tests. CE = Consumer-employer. SF-12 = 12-item short form health survey. ^a^ Sum of nine items rated on a five-point scale, responses range from 1 (strongly disagree) to 5 (strongly agree). ^b^ Six-point frequency scales, responses ranged from 0 (never) to 5 (5+ times). ^c^ Items related to sugary snacks, drinks, fast food, and meals from home were reported on 10 frequency intervals: 1, never | 2, 1–3 times per month | 3, 1–2 times per week | 4, 3–4 times per week | 5, 5–6 times per week | 6, Once per day | 7, 2 times per day | 8, 3 times per day | 9, 4 times per day | 10, 5 or more times per day. Thus, a mean score of 3 would equal the behavior occurring 1–2 times per week. ^d^ Mean of four items asking about days per week with 30 min of different moderate-to-vigorous physical activities. Eight-point response scale ranged from 0 (none) to 7 (daily).

**Table 4:** Worker evaluations for COMPASS groups in the Oregon Home Care Commission training system: Mean (*SD*) ratings for quantitative questions in the original evaluation format.

Question	Meeting Number (Sample Size)								
	Unknown (*n* = 33)	1 (*n* = 26)	2 (*n* = 41–42)	3 (*n* = 30)	4 (*n* = 14)	5 (*n* = 15–16)	6 (*n* = 27–28)	7 (*n* = 39–40)	Overall (*n* = 229)
The training met my expectations and needs	4.69 (0.66)	4.36 (0.63)	4.41 (0.59)	4.60 (0.56)	4.64 (0.63)	4.75 (0.44)	4.75 (0.44)	4.80 (0.46)	4.67 (0.55)
The information will be useful in my work	4.67 (0.65)	4.36 (0.74)	4.69 (0.56)	4.67 (0.61)	4.79 (0.43)	4.80 (0.41)	4.79 (0.42)	4.90 (0.38)	4.74 (0.53)
Info. was presented in ways that facilitated learning	4.64 (0.65)	4.43 (0.85)	4.67 (0.57)	4.67 (0.48)	4.64 (0.63)	4.88 (0.34)	4.75 (0.44)	4.76 (0.48)	4.70 (0.55)
The trainer/facilitator was well prepared and organized	4.45 (0.79)	4.36 (0.74)	4.57 (0.59)	4.73 (0.45)	4.79 (0.43)	4.81 (0.40)	4.75 (0.44)	4.83 (0.45)	4.68 (0.56)
The trainer/facilitator communicated effectively	4.48 (0.76)	4.50 (0.65)	4.62 (0.58)	4.77 (0.43)	4.86 (0.36)	4.81 (0.40)	4.75 (0.44)	4.85 (0.43)	4.72 (0.53)
The handouts (or written materials) are helpful	4.57 (0.68)	4.54 (0.88)	4.67 (0.48)	4.66 (0.55)	4.57 (0.65)	4.88 (0.34)	4.74 (0.44)	4.86 (0.41)	4.71 (0.53)
I would recommend this training to others	4.70 (0.64)	4.46 (0.88)	4.69 (0.56)	4.71 (0.53)	4.71 (0.47)	4.88 (0.34)	4.75 (0.44)	4.90 (0.38)	4.75 (0.53)

Note: “Info.” = information. Rating scale was 1 = poor, 2 = fair, 3 = average, 4 = good, and 5 = excellent.

**Table 5:** Worker evaluations for COMPASS groups in the Oregon Home Care Commission training system: Mean (*SD*) ratings for quantitative questions in the revised/new format.

Question	Overall (*n* = 36)
The training met my expectations and needs	3.58 (0.50)
The information will be useful in my work	3.56 (0.50)
Information was presented in ways that facilitated learning	3.61 (0.50)
The trainer/facilitator was well prepared and organized	3.63 (0.48)
The trainer/facilitator communicated effectively	3.58 (0.50)
The handouts (or written materials) are helpful	3.55 (0.51)
I would recommend this training to others	3.62 (0.65)
I enjoyed this training	3.58 (0.66)
I will do something new, different, or better in order to be safer at work because of this training	3.53 (0.51)
I will do something new, different, or better in order to improve my health and well-being because of this training	3.66 (0.48)
I will do something new, different, or better in order to improve my consumer-employers’ health and well-being because of this training	3.60 (0.50)

Note: rating scale was 1 = strongly disagree, 2 = disagree, 3 = agree, 4 = strongly agree, and also a “does not apply” option. If participants marked “does not apply” this was coded as missing data and was not included in calculations.
